# Caldendrin Is a Repressor of PIEZO2 Channels and Touch Sensation in Mice

**DOI:** 10.1523/JNEUROSCI.1402-23.2023

**Published:** 2024-03-06

**Authors:** Josue A. Lopez, Luis O. Romero, Wai-Lin Kaung, J. Wesley Maddox, Valeria Vásquez, Amy Lee

**Affiliations:** ^1^Department of Neuroscience and Center for Learning and Memory, University of Texas-Austin, Austin 78712, Texas; ^2^Department of Physiology, The University of Tennessee Health Science Center, Memphis 38163, Tennessee; ^3^Integrated Biomedical Sciences Graduate Program, College of Graduate Health Sciences, Memphis 38163, Tennessee

**Keywords:** calcium, dorsal root ganglion, ion channel, mechanosensation, PIEZO2, touch sensation

## Abstract

The sense of touch is crucial for cognitive, emotional, and social development and relies on mechanically activated (MA) ion channels that transduce force into an electrical signal. Despite advances in the molecular characterization of these channels, the physiological factors that control their activity are poorly understood. Here, we used behavioral assays, electrophysiological recordings, and various mouse strains (males and females analyzed separately) to investigate the role of the calmodulin-like Ca^2+^ sensor, caldendrin, as a key regulator of MA channels and their roles in touch sensation. In mice lacking caldendrin (*Cabp1* KO), heightened responses to tactile stimuli correlate with enlarged MA currents with lower mechanical thresholds in dorsal root ganglion neurons (DRGNs). The expression pattern of caldendrin in the DRG parallels that of the major MA channel required for touch sensation, PIEZO2. In transfected cells, caldendrin interacts with and inhibits the activity of PIEZO2 in a manner that requires an alternatively spliced sequence in the N-terminal domain of caldendrin. Moreover, targeted genetic deletion of caldendrin in *Piezo2*-expressing DRGNs phenocopies the tactile hypersensitivity of complete *Cabp1* KO mice. We conclude that caldendrin is an endogenous repressor of PIEZO2 channels and their contributions to touch sensation in DRGNs.

## Significance Statement

Our sense of touch allows us to discriminate shapes and textures, as well as influences learning, emotion, and social relationships. Touch sensation relies on the activity of mechanically activated ion channels in peripheral sensory neurons, but the molecular mechanisms that regulate this process are poorly understood. Our study identified a requirement for the neuronal Ca^2+^ sensor caldendrin as a key regulator of PIEZO2 mechanically activated channels and their roles in touch sensation. Our findings establish a molecular pathway that could be targeted in novel therapies for conditions that involve aberrant responses to touch such as autism spectrum disorder and neuropathic pain.

## Introduction

Somatosensory neurons in the dorsal root ganglion (DRG) convey information needed for appropriate behavioral and emotional responses to the environment. A subset of DRG neurons (DRGNs) exhibit molecular and structural specializations to accurately encode mechanical signals, such as those arising from touch, pressure, and vibration (Handler and Ginty, 2021). These neurons enable the discrimination of texture and shape, as well as innocuous from harmful tactile stimuli, and their function can change with aging (Feng et al., 2018), injury (Zhu and Henry, 2012), and neurodevelopmental disorders (Orefice et al., 2019). It is well-established that touch sensation relies on mechanically activated (MA) ion channels, which transduce force into an electrical response (Delmas and Coste, 2013; Ikeda et al., 2014; Maksimovic et al., 2014; Ranade et al., 2014; Handler and Ginty, 2021). However, the factors that regulate MA channels and their contributions to mechanosensation are poorly understood.

Caldendrin is a member of the superfamily of EF-hand Ca^2+^ binding proteins and is highly homologous to calmodulin (CaM) (Seidenbecher et al., 1998; Haeseleer et al., 2000). Caldendrin and a shorter splice variant, Ca^2+^-binding protein 1 (CaBP1), are encoded by the *Cabp1* gene. Like CaM, caldendrin/CaBP1 bind to a variety of ion channels and, in some cases, antagonize the effects of CaM on channel function (Zhou et al., 2004; Kinoshita-Kawada et al., 2005; Tippens and Lee, 2007; Findeisen et al., 2013; Li et al., 2013). Unlike CaM, caldendrin is expressed primarily in neuronal cell types (Kim et al., 2014) and is particularly abundant in sensory neurons in the inner ear and DRG (Yang et al., 2016; Lopez et al., 2023). Caldendrin is the main *Cabp1* splice variant expressed within the DRG and is highly expressed in large myelinated DRGNs (Lopez et al., 2023), which are involved in transmitting information about innocuous touch (Abraira and Ginty, 2013). To test the hypothesis that caldendrin regulates the properties of MA channels in DRGNs and their involvement in touch sensation, we used global and conditional *Cabp1* knock-out (KO) mouse strains in concert with electrophysiological, behavioral, and biochemical strategies. Our findings support a role for caldendrin as a negative regulator of PIEZO2 in mechanosensory DRGNs that mediate the sense of touch.

## Materials and Methods

### Animals

All experiments were performed in accordance with guidelines set by the Institutional Animal Care and Use Committee at the University of Iowa and University of Texas-Austin. Mice were housed under a standard 12-h light/dark cycle with access to food and water ad libitum. The *Cabp1* KO mouse strain (RRID: MGI: 5780462) was described previously (Kim et al., 2014) and maintained on a C57BL/6J background. WT mice were age- and sex-matched C57BL/6J mice. Mice expressing Cre and a PIEZO2 fusion with enhanced green fluorescent protein (PIEZO2-GFP) under the endogenous Piezo2 promoter (*Piezo2*^Cre^) (Woo et al., 2015) were obtained from Jackson Laboratories (RRID:IMSR_JAX:027719). Floxed *CaBP1* mice (*Cabp1*^fl/fl^) mice were obtained from MRC Harwell Institute (EM:05847) and bred with *Piezo2*^Cre^ mice to generate the conditional *Cabp1* KO mouse strain (*Piezo2*^Cre^/*Cabp1*^fl/fl^).

### Immunofluorescence and confocal microscopy

Mice were anesthetized in a narcosis chamber with isoflurane and euthanatized by cervical dislocation and decapitation. Lumbar DRGs (L4–L6) were harvested from male or female mice (6–10 weeks old) and fixed using 4% paraformaldehyde in phosphate-buffered saline (PBS) overnight at 4°C. Fixed DRGs were then infused with 15% sucrose in PBS 24 h at 4°C. The fixed tissue was molded in Optimal Cutting Temperature compound (Sakura Finetek) and 15-mm-thick sections were obtained using a cryostat (Leica). Sections were mounted on charged slides and dried on a slide warmer for 5 min. Sections were then subject to rinsing, blocking, and antibody incubating at room temperature (RT). After rinsing in PBS and incubating in blocking buffer (10% normal goat serum in 0.2% Triton X-100, in PBS) for 30 min, the sections were incubated for 1 h in the following primary antibodies diluted 1:1,000 in blocking buffer: chicken polyclonal anti-NF200 (RRID: AB_2313552, Aves); rabbit polyclonal anti-CaBP1/caldendrin (UW72 (Haeseleer et al., 2000); these antibodies do not produce immunoreactivity in tissues of *Cabp1* KO mice (Kim et al., 2014)); mouse monoclonal anti-parvalbumin (Sigma-Aldrich Cat# P3088, RRID:AB_477329); mouse monoclonal anti-calcitonin gene-related peptide (CGRP) (Sigma-Aldrich Cat# C9487, RRID:AB_1078377); mouse monoclonal anti-Green Fluorescent Protein GFP (Novus Cat# NB110-40670, RRID:AB_714984). Sections were washed three times with PBS for 10 min followed by incubation with secondary antibodies diluted 1:500 in blocking buffer: goat anti-Chicken Alexa Fluor 546 (RRID: AB_2534097, Thermofisher), goat anti-Chicken Alexa Fluor 647 (RRID:AB_2535866, Thermofisher), goat anti-Rabbit Alexa Fluor 488 (RRID:AB_2534096, Thermofisher), goat anti-Rabbit Alexa Fluor 555 (RRID:AB_2535849, Thermofisher), goat anti-Mouse Alexa Fluor 488 (RRID:AB 2576208, Thermofisher), goat anti-Mouse IgG2a, Alexa Fluor 488 (RRID:AB_2535771, Thermofisher) and goat anti-Mouse IgG1, CF647 (RRID:AB 10853957, Biotium). Isolectin B4 (IB4), FITC conjugate (Sigma-Aldrich Cat# L2895) was used to label small DRGNs. Sections were then washed three times with PBS for 10 min each. Coverslips (#1.5) were then mounted using Fluoromount-G (Southernbiotech) and allowed to cure for 24 h in the dark before imaging. DRGs from at least 3 mice were processed in 3 independent experiments. At least 3 sections per animal were analyzed using an Olympus Fluoview 3000 confocal microscope equipped with an UPlanXApo 20× objective. Images were captured using Olympus FLUOVIEW software (RRID: SCR_014215). Acquisition settings were standardized and optimized using the Hi-Low saturation mask to prevent signal saturation prior to collecting z-stacks. All confocal images are presented as the maximum Z-projections.

For quantifying the number of NF200-positive DRGNs expressing either CaBP1, PIEZO2-GFP, or CaBP1 and PIEZO2-GFP, confocal Z-stack images of DRGs from CaBP1 KO and PIEZO2-GFP mice immunolabeled for CaBP1, PIEZO2-GFP and NF200 were processed using Amira software (Thermo Fisher). For each Z-stack, the NF200 channel was used to create a binary mask. This binary mask was then used to subtract any signal in the CaBP1 and PIEZO2-GFP channels that was not within NF200 immunofluorescence. The number of DRGN somas of the subtracted images with immunofluorescence for either CaBP1, PIEZO2-GFP, or CaBP1 and PIEZO2-GFP were counted using ImageJ (NIH).

### Behavioral assays

All behavioral tests and analyses were conducted by an experimenter blind to genotype. Von Frey, Hargreaves, and cold plantar assays were performed on the same cohorts of male or female mice (8–12-week-old). Each assay was replicated at least three times with the same cohort of mice. Prior to the behavioral assays, mice were handled and adapted to the environment for at least 3 consecutive days. For each assay, animals were acclimatized to the testing room for 30 min prior to the experiment on the day of testing. This was accomplished by placing the mice on platforms with Plexiglass covers for at least 30 min.

The von Frey assay was conducted utilizing Ugo Basile Dynamic Plantar Aesthesiometer (RRID:SCR_021751) as previously described (Mickle et al., 2015). The assay was conducted using 8 von Frey filaments (0.04, 0.07, 0.16, 0.4, 0.6, 1.0,1.4, 2 g; Stoelting Co, Wood Dale, IL) which were applied to the plantar surface of each hind paw 5 times starting from the lowest filament strength (0.04 g) and moving up to the highest (2 g). The withdrawal responses were recorded based on the number of times the mouse withdrew the paw out of 5 trials for each filament applied, which was represented as % withdrawal response.

The Hargreaves and cold plantar assays were conducted on the plantar test apparatus (IITC Life Sciences, Woodland Hills, CA) as previously described (Mickle et al., 2015). In the Hargreaves assay, the heat stimulus was applied using a 100-W projector lamp (50°C), with an aperture diameter of 6 mm, applied from underneath the glass floor (maintained at 30°C) onto the middle of the tori of each hind paw. The latency of paw withdrawal was recorded with a cutoff value of 25 s. At least 7 min was allowed between testing the paws on a single mouse, and an interval of at least 15 min was allowed between trials on any single paw for both Hargreaves and cold plantar assay. Each mouse was tested at least 2 times per paw to obtain the average for paw withdrawal latency.

The cold plantar assay utilizes a cold stimulus delivered by applying a cut-off syringe filled with crushed dry ice (for temperature ranges of 5–12°C) to the glass underneath the paw as previously described in (Brenner et al., 2012). The paw withdrawal latency was measured using the same setup as for the Hargreaves assay. Each mouse was tested at least 4 times per paw and a maximum time of 20 s was set up as cutoff to avoid potential tissue damage.

The tape assay was modified from a protocol described previously (Ranade et al., 2014; Murthy et al., 2018; Michel et al., 2020). Cohorts of female mice (12 weeks) were acclimatized to the assay room for 30 min 2 consecutive days before, as well as on, the day of the experiment. For the assay, the mice were separated into individual arenas and allowed to explore for 5 min. To test mechanical sensitivity on the hairy skin, a circular adhesive tape (white, 10 mm, Tough-Spots) was placed on the upper back of mice. The mice were then returned to their respective arenas and the following tape-directed responses were recorded as a “bout”: wet-dog shake, trying to reach tape with snout, bursts of biting tape, bursts of pulling tape with front paws, bursts of scratching tape with hind leg. The behaviors were recorded with an area scan camera (Basler acA1300 – 60gm) and EthoVision XT16 software. Recording was stopped when a mouse successfully managed to remove the tape or at the end of the 5-min trial. The following parameters were analyzed: the interval between placing the animal in the cage and the first bout (response latency), total number of bouts throughout the trial, bouts per minute and tape riddance (% of cohort). Data analysis and scoring was done manually by researchers blinded to mouse genotype.

The hanging-wire assay and the inverted screen test was modified from a previously described protocol (Florez-Paz et al., 2016; Ma et al., 2023). Cohorts of WT and *Ca*bp1 KO; male and female mice (12–13 weeks) were acclimatized to the assay room for 15 min before the experiments. For the hanging-wire assay, a 90 cm long, 2-mm-thick metal wire was suspended 40 cm above soft bedding and secured between two vertical poles. The wire was adjusted to minimize slack and prevent vibrations during an assay trial. The suspension time for each mouse was measured in 3 trials with 1-minute recovery periods and the average across the 3 trials was reported. Trials were concluded after 30 s, regardless of whether a mouse had yet to fall from the wire. For the inverted screen test, the mice were placed individually on a 22 cm × 30 cm metal grid screen which was then rotated 180 degrees to position the mice 40 cm above soft bedding. Suspension time was recorded in 6 trials, with 1-minute recovery periods. If a mouse climbed to the top, the screen was rotated again to ensure the animal remained inverted. The average time across all trials was reported, with a 90 s trial cutoff.

### Western blot analysis of mouse DRGs

Five lumbar DRGs were harvested from each mouse (4–10-week-old males or females), flash frozen in liquid nitrogen, and lysed in N-PER Neuronal Protein Extraction Reagent (Thermo Fisher Cat# 87792) with Protease Inhibitor Cocktail and 1 mM PMSF. Nupage LDS Sample Buffer (4×) (Thermo Fisher Cat# NP0007) was added to lysates which were then incubated at 65°C for 10 min. The samples were loaded into a 10 well 4–20% Tris-Glycine gel (Invitrogen Cat# XP04200BOX), run in Tris-Glycine SDS Running buffer (Novex Life technologies Cat# LC2675), and transferred overnight using Tris-Glycine Transfer Buffer (Novex Life technologies Cat# LC3675). Blots were incubated in blocking buffer consisting of 5% fat free milk in tris-buffered saline with 0.05% Tween (Thermo Fisher Cat# J77500.K8; TBST) for 30 min at RT prior to incubation for 2 h or overnight in primary antibodies diluted in blocking buffer at 4°C: rabbit anti-UW72 1:1,000 or mouse anti- β-Actin 1:3,000 (Sigma-Aldrich Cat# A5316, RRID:AB_476743). After primary antibody labeling, the blots were wash 4 times with TBST and incubated with secondary HRP antibody ECL anti- rabbit (1:4,000, Cytiva Cat# GENA934, RRID:AB_2722659) and ECL anti-mouse IgG (1:3,000, Cytiva Cat# NA9310-1 ml, RRID:AB_772193) for 1 h at RT. The blots were then washed 4 times with TBST and developed with Chemiluminescent reagent (Thermo Fisher Cat# 34580) prior to exposure in an iBright 750 imager (Thermo scientific). Densitometric analyses were done using Image Studio Lite software. Following subtraction of background signals, the signal corresponding to GFP-PIEZO2 and caldendrin was normalized to that for β-Actin.

### Transfection of N2A cells

Mouse neuro-2a (N2A; CCL-131) cell line was obtained from the American Type Culture Collection (ATCC). PIEZO1 knock-out mouse N2A (Piezo1^−/−^ N2A) cells were a gift from Dr. Gary R. Lewin (Moroni et al., 2018). N2A and Piezo1^−/−^ N2A cells were cultured in Dulbecco's Modified Eagle's medium (DMEM), 5% penicillin–streptomycin, and 10% fetal bovine serum (FBS) and maintained at 37°C, with 95% relative humidity and 5% CO_2_. N2A and Piezo1^−/−^ N2A cells were transfected using Lipofectamine 2000 (ThermoFisher Scientific), according to the manufacturer's instructions. Piezo1^−/−^ N2A cells were grown in 6-well plates to 80% confluency and co-transfected with cDNAs encoding: mouse PIEZO2 variant 14-pcDNA3.1(+) (0.25 µg/ml), GFP-pMO (a pcDNA3.1-based vector with the 5' and 3' untranslated regions of the β-globin gene, 50 ng/ml), and GFP-tagged mouse caldendrin variants (GFP-Caldendrin-L or GFP-Caldendrin-S, Genbank # KJ364651.1, 150 ng/ml) or CaBP1 (GFP-CaBP1-S, Genbank # NM_013879.2, 150 ng/ml). N2A cells were transfected in the same conditions and cDNAs omitting the PIEZO2 construct. The GFP-caldendrin and CaBP1 constructs were generated by cloning with C-terminal EGFP tag into HindIII, NotI sites of pRNA2 vector. After 48 h, the cells were plated on Poly-L-Lysine (Sigma-Aldrich)-treated glass coverslips in 24-well plates for electrophysiological recordings.

### Co-immunoprecipitation (Co-IP) assay

Human embryonic kidney cells transformed with SV40T-antigen (HEK293T, ATCC CRL-3216) were maintained in DMEM supplemented with 10% FBS and 5% penicillin–streptomycin in a humidified incubator with 5% CO_2_ at 37°C. The cells were plated in 35 mm dishes and transfected using Lipofectamine 3000 (Life Technologies) according to the manufacturer's instructions. 35 mm plates of HEK293T cells were transfected with cDNAs encoding: HA-PIEZO2 variant 14-pcDNA3.1(+) (1.5 µg) (Szczot et al., 2017), and GFP (0.5 µg) or GFP-Caldendrin (0.5 µg) or GFP-CaBP1-S (0.5 µg) described for N2A transfections. Negative control groups were transfected with GFP-caldendrin or GFP-CaBP1-S alone.

Twenty-four hours later, transfected cells were lysed in lysis buffer (50 mM Tris-HCl pH7.4; 150 mM NaCl; 0.5% Triton X100; 0.5% n-Dodecyl-β-Maltoside detergent, Thermo Fisher Cat# 89902), Protease Inhibitor Cocktail (Roche complete, EDTA-free, Cat# 05056489001) and 1 mM phenylmethylsulfonyl fluoride (RPI, SKU# P20270). Plastic pestles were used to homogenize the pellet in a 1.5 ml microfuge tube and lysates were incubated on ice for 10 min. Lysates were cleared by spinning down samples at 14,000 × *g* at 4°C for 10 min. The lysate was collected and 80% of the lysate was incubated in 30 µl of bed volume of Pierce Anti-HA Magnetic Beads (Thermo Fisher Cat# 88836) per sample and incubated at 4°C for 1 h with rotation. The beads were pelleted using a magnet washed three times with 1 ml of PBS prior to resuspending in 50 µl of 1× Nupage LDS Sample Buffer and incubating at 65°C for 10 min prior. The samples were processed for western blot using anti-rabbit UW72 1:1,000, anti-rabbit GFP 1:1,000 (Thermo Fisher Scientific Cat# A-11122, RRID:AB_221569), and goat anti-mouse β-Actin 1:3,000 (Sigma-Aldrich Cat# A5316, RRID:AB_476743). Western blot detection and densitometry was performed as described for DRG lysates. For quantitative analysis, background-subtracted signals corresponding to GFP-caldendrin or GFP-CaBP1-S in the total input sample was divided by that for β-Actin. This value was used to normalize the signals corresponding to the GFP-tagged proteins Co-IP'ed with the anti-HA antibodies.

### Preparation of mouse DRGNs for electrophysiology

At the University of Texas-Austin, WT and *Cabp1* KO mice were anesthetized in a narcosis chamber with isoflurane and euthanatized by cervical dislocation and decapitation. 15–20 DRGs were harvested from 3 different WT and Cabp1 KO female mice (4–6-weeks old) and stored in 2 ml of Neurobasal media supplemented with 2.5% L-glutamine (ThermoFisher, Cat #25030081), 2% N-21 (Millipore Sigma, Cat #SCM081), and penicillin–streptomycin (ThermoFisher, Gibco, Cat #15140122) and shipped over night with ice packs to the University of Tennessee Health Sciences Center. Upon receipt, the DRGs were incubated with 1 mg/ml collagenase B (Sigma) in HBSS at 37°C and 5% CO_2_ and then, after 1 h, were dissociated in a medium without serum. The cell suspension solution was centrifuged for 8 min at 800 × *g*. The pellet was resuspended in DMEM (Gibco) complete media containing 1% penicillin–streptomycin (Gibco), 1% MEM vitamin solution (Gibco), 1% L-glutamine (Gibco), and 10% horse serum (Gibco). Cells were plated on Poly-L-Lysine (Sigma-Aldrich)-treated glass coverslips in 24-well plates. All mouse DRGNs were kept at 37°C, with 95% relative humidity and 5% CO_2_. Cells were used in experiments after 24 h.

### Electrophysiological analysis of MA currents

Patch-clamp recordings were performed on cells plated on glass coverslips. For whole-cell recordings of mechano-activated currents in the voltage-clamp mode, the bath solution contained 140 mM NaCl, 6 mM KCl, 2 mM CaCl_2_, 1 mM MgCl_2_, 10 mM glucose, and 10 mM HEPES (pH 7.4), while the pipette solution contained 140 mM KCl, 6 mM NaCl, 2 mM CaCl_2_, 1 mM MgCl_2_, 10 mM glucose, and 10 mM HEPES (pH 7.4). Pipettes were fabricated from borosilicate glass capillaries (Sutter Instruments) and fire-polished to a resistance between 3 and 5 MΩ before use. Mechanical stimulation was performed during voltage-clamp recordings with cells held at −60 mV. Recordings were sampled at 100 kHz and low pass filtered at 10 kHz using a MultiClamp 700 B amplifier and Clampex (v10.4.2.0; Molecular Devices, LLC). Leak currents before mechanical stimulation were subtracted offline from the current traces and data were digitally filtered at 2 kHz with ClampFit (v10.4.2.0; Molecular Devices, LLC). Recordings with leak currents >200 pA, with access resistance >10 MΩ, and cells with giga-seals that did not withstand at least six consecutive steps of mechanical stimulation were excluded from analyses.

For indentation assays, DRGNs, N2A, and transfected Piezo1^−/−^ N2A cells were mechanically stimulated with a heat-polished blunt glass pipette (3–4 µm) driven by a piezo servo controller (E625, Physik Instrumente). The blunt pipette was mounted on a micromanipulator at an ∼45° angle and positioned 3–4 µm above the cells. Displacement measurements were obtained with a square-pulse protocol consisting of 1 µm incremental indentation steps, each lasting 200 ms with a 2 ms ramp in 10 s intervals. The threshold of mechano-activated currents for each experiment was defined as the indentation step that evoked the first current deflection from the baseline. Only cells that did not detach throughout stimulation protocols were included in the analyses. The piezo servo controller was automated, using a MultiClamp 700B amplifier, through Clampex (Molecular Devices, LLC).

Data were plotted using OriginPro (2018 v:b9.51.195; OriginLab Corp.). The time constant of inactivation *τ* was obtained by fitting a single exponential function (X) between the peak value of the current at the end of the stimulus:

$$f_{\left(x\right)}=\overset{n}{\underset{i=1}{\sum }}\;A_{i}*e^{\frac{-t}{T_{i}}}+C$$

where *A* = amplitude, *τ* = time constant, and the constant y-offset *C* for each component *i*.

All boxplots show mean (square), median (bisecting line), bounds of box (75th to 25th percentiles), and outlier range with 1.5 coefficient (whiskers).

### Experimental design and statistical analyses

No statistical method was used to predetermine the sample size. The experiments were not randomized. For electrophysiology, the investigator was blind to genotype and treatment. All attempts at replication were successful. Experiments were performed at least three times on different days with different/independent preparations. Statistical analyses for electrophysiology were performed using GraphPad InStat software (version 3.10; GraphPad Software Inc.) and OriginPro. We used the Kolmogorov and Smirnov method to determine normality of data, as well as Bartlett's test to determine differences between standard deviations. Specific tests are described in figure legends.

For behavioral studies, GraphPad Prism 9 software (RRID: SCR_002798, GraphPad Software) was used to generate graphs and statistical analysis. All data sets were first tested for normality by Shapiro–Wilk normality test. When data sets were normally distributed, the data was analyzed by unpaired *t* test or ANOVA with Tukey's multiple comparison test with a single pool variance. If the data was not normally distributed, the data was analyzed by Mann–Whitney test. For two-way ANOVA tests with main effects only, Sidak's multiple comparisons test was performed with individual variances calculated for each comparison. Data was presented as mean and SEM and *p *≤ 0.05 was considered statistically significant.

### Data availability

Data supporting the findings of this manuscript are available from the corresponding author upon request. The source data for western blot images can be accessed in the Figshare data repository: https://figshare.com/s/a9a0635790418de1cbe9.

## Results

### Caldendrin is expressed in a subset of DRGNs involved in touch sensation

Mechanically responsive DRGNs are highly heterogeneous and specialized for responses to a wide range of physical stimuli. Those that innervate the skin are classified as low-threshold mechanoreceptors (LTMRs) or high-threshold mechanoreceptors (HTMRs) based on their responses to stimuli that are innocuous or harmful, respectively. Ab-LTMRs respond to indentation, stretch, and vibration, whereas Ad- and C-LTMRs can be activated by weak mechanical stimuli and phasic temperature changes (Abraira and Ginty, 2013). Proprioceptive DRGNs innervate muscle, spindles, tendons, and skin, and respond to mechanical stimuli needed to convey the sense of body and limb position (Proske and Gandevia, 2012).

**Figure 1.**
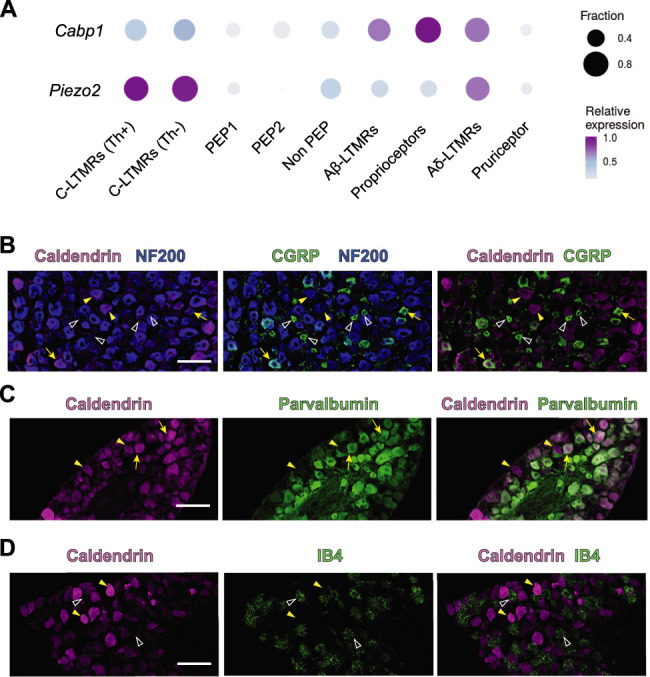
Caldendrin is expressed in DRGNs involved in mechanical sensation. ***A***, Analysis of *Cabp1* and *Piezo2* mRNA in specific DRGN cell types based on scRNA dataset from Renthal et al. (2020). For each gene, the size and color of each circle represents the fraction of cells expressing and the expression levels, respectively. ***B–D***, Confocal micrographs of cryosections of mouse DRGs labeled with antibodies against caldendrin and antibodies against NF200 and CGRP (***B***), parvalbumin (***C***), or IB4 (***D***). In ***B***, caldendrin labeling is mostly found in NF200 + neurons (arrowheads) and large (arrows) but not small (open arrowheads) CGRP + neurons. In ***C***, caldendrin labeling is found in parvalbumin positive (arrows) and negative neurons (arrowheads). In ***D***, caldendrin labeling (filled arrowheads) is found in neurons other than those labeled by IB4 (open arrowheads). Results represent 3 independent experiments (*N* = 3 mice). Scale bar, 100 μm.

To gain insights into the somatosensory function of caldendrin, we analyzed the expression of CaBP1 in specific classes of DRGNs using a single cell (sc)RNA-seq dataset (GSE154659) described previously (Renthal et al., 2020). We previously showed that most (∼92%) of DRGNs that express caldendrin also express neurofilament 200 (NF200) (Lopez et al., 2023)—a marker of mechanically sensitive DRGNs that includes A-b LTMRs, A-d LTMRs, and proprioceptors (Kupari et al., 2021). Consistent with these findings, the scRNAseq analysis indicated highest expression of *Cabp1* mRNA in DRGNs that express *NF200* mRNA. Lower levels of *Cabp1* expression typify C-fiber DRGNs, such as C-LTMRs, nociceptors, and pruriceptors (Fig. 1*A*). The expression of caldendrin in A-b LTMRs, A-d LTMRs, and proprioceptors was confirmed by double-labeling with antibodies against caldendrin and those recognizing NF200 or parvalbumin (de Nooij et al., 2013); many but not all NF200-positive DRGNs also exhibited caldendrin labeling (Fig. 1*B,C*). To identify expression in peptidergic and/or nonpeptidergic nociceptors, we labeled with antibodies against calcitonin gene related peptide (CGRP) and with fluorescently tagged plant lectin, isolectin B4 (IB4), respectively (Dubin and Patapoutian, 2010). While largely absent from IB4 positive and small CGRP-positive DRGNs, caldendrin was present in large CGRP/NF200 positive DRGNs that likely represent polymodal C-LTMRs (Kestell et al., 2015; Fig. 1*B,D*). These results show that caldendrin typifies a variety of mechanically sensitive DRGNs including those known to respond to tactile stimuli. This pattern of expression resembles that for the mechanically gated ion channel PIEZO2, which mediates touch sensation and mechanical allodynia in mice (Ranade et al., 2014; Murthy et al., 2018).

### Mechanical sensitivity is potentiated in Cabp1 KO mice

**Figure 2.**
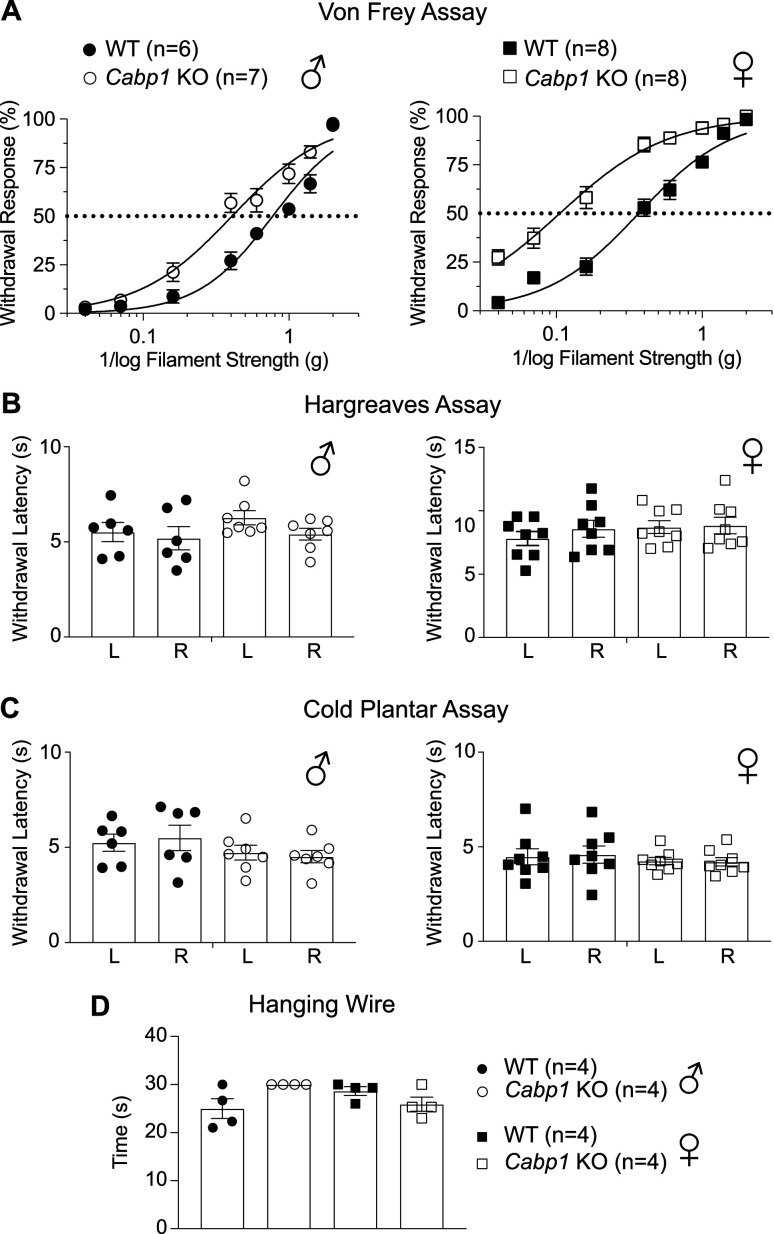
*Cabp1* KO mice have increased mechanical sensitivity but normal temperature sensation. ***A–D***, WT (filled symbols) and *Cabp1* KO (open symbols) mice (Left panels: males, circles; right panels: females, squares) were subject to assays measuring sensitivity to mechanical (von Frey assay, ***A***) or thermal (Hargreaves assay, ***B***; Cold plantar assay, ***C***) stimuli, or proprioception (Hanging wire assay, ***D***). In ***A***, % Withdrawal response (relative to 5 stimulus trials) is plotted against 1/log filament strength. Symbols/bars represent mean ± SEM. Dotted line represents 50% withdrawal response. Smooth lines represent fit by non-linear regression. By 2 way-ANOVA, datasets were significantly different for WT and *Cabp1* KO mice. For males: *F*_(7,77)_ = 6.114, *p* < 0.0001; for females: *F*_(7,98)_ = 10.42, *p* < 0.0001. In ***B,C***, withdrawal latencies are plotted for left (L) and right (R) paws for Hargreaves (***B***) and cold plantar (***C***) assays. Bars represent mean ± SEM. By one-way ANOVA, there were no significant differences in WT and *Cabp1* KO mice. In ***B***, *F*_(3,22)_ = 1.144, *p* = 0.3533 for males, *F*_(3,28)_ = 0.5969, *p* = 0.6223 for females. In ***C***, *F*_(3,22)_ = 0.9752, *p* = 0.4223 for males, *F*_(3,28)_ = 0.2824, *p* = 0.8376 for females. The same cohorts were used in all 3 assays. In ***D***, there was no significant difference in time remaining on the wire for WT and *Cabp1* KO males or females (*H*_(3) _= 6.391, *p* = 0.0872 by Kruskal-Wallis test).

Based on the expression pattern of caldendrin in DRGNs, we hypothesized that loss of caldendrin could manifest as a defect in responsiveness to mechanical stimuli. To test this hypothesis, we compared the performance of wild-type (WT) and *Cabp1* KO mice in a series of behavioral assays. To characterize cutaneous sensitivity, we used the von Frey assay which measures paw withdrawal responses to filaments of increasing force. Both male and female *Cabp1* KO mice were more responsive than their WT counterparts to a wide range of filament strengths (Fig. 2*A*). The force eliciting the half-maximal response was significantly lower for *Cabp1* KO mice than WT mice for males (mean ± SEM: 0.44 ± 0.07 g for KO vs 0.78 ± 0.06 g for WT, *t*_(11)_ = 3.8, *p* = 0.003 by unpaired *t* test) and females (median, IQR: 0.12,0.28–0.42 g for KO vs 0.36, 0.27–0.43 for WT, *U* = 0, *p* = 0.0002 by Mann–Whitney *U* test). Given the limited expression of caldendrin in small-diameter, NF200-negative DRGNs (Fig. 1*A*), such as those that sense heat and cold (Peier et al., 2002; McCoy et al., 2013), we did not expect that temperature sensitivity would be affected in *Cabp1* KO mice. Consistent with this prediction, WT and *Cabp1* KO mice did not differ in paw withdrawal responses in the Hargreaves and cold plantar assays (Fig. 2*B,C*). Given the expression of caldendrin in parvalbumin-positive DRGNs (Fig. 1*A,C*), we also tested behaviors that would indicate a proprioceptive deficit (Florez-Paz et al., 2016; Espino et al., 2022; Ma et al., 2023). However, *Cabp1* KO mice showed normal limb positioning and paw clasping behaviors and remained for same duration as WT mice while suspended on an inverted grid (data not shown) and wire (Fig. 2*D*). Collectively, these results support a role for caldendrin in dampening responsiveness to mechanical but not thermal or proprioceptive stimuli.

**Figure 3.**
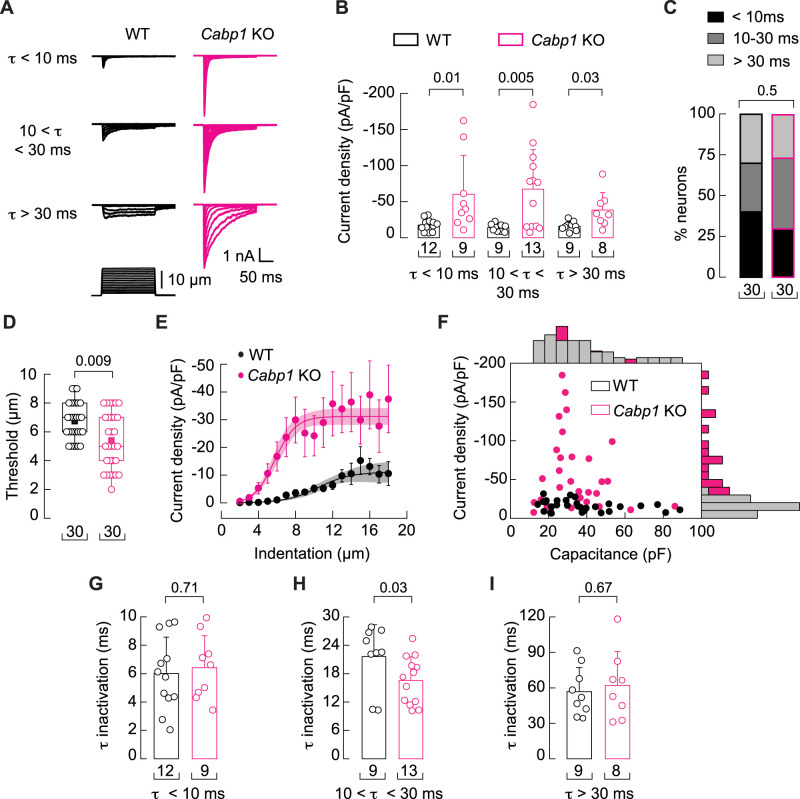
MA currents are potentiated in *Cabp1* KO DRGNs. MA currents were evoked by mechanical indentation at a voltage of −60 mV in whole-cell patch clamp recordings of DRGNs isolated from WT and *Cabp1* KO female mice. ***A***, Representative traces corresponding to rapidly (top traces; *t* < 10 ms), intermediate (middle traces; 10 < *t* < 30 ms), and slowly (lower traces; *t* > 30 ms) inactivating currents. Mechanical stimulus protocol is shown below. ***B***, Peak current densities elicited by maximum indentation for the different classes of MA currents shown in ***A***. Symbols and bars represent individual cells and mean + SD, respectively. ***C***, Proportions of neurons classified by MA current inactivation kinetics. ***D***, Displacement thresholds required to elicit MA currents. Boxplots show mean (square), median (bisecting line), bounds of box (75th to 25th percentiles), outlier range with 1.5 coefficient (whiskers), and minimum and maximum data points. ***E***, Current densities elicited by maximum displacement. Symbols and bars are mean ± SEM, respectively. ***F***, Scatter plot of current density versus capacitance of WT and *Cabp1* KO DRGNs. Top and right display the corresponding marginal histograms. ***G–I***, Time constants of inactivation (*t*) were measured upon maximum indentations for rapidly adapting (***G***), intermediately adapting (***H***), and slowly adapting (***I***) MA currents. Symbols and bars represent individual cells and mean + SD, respectively. In ***B–I***, *p* values from post hoc tests for WT versus *Cabp1* KO data are shown and determined by Kruskal-Wallis (*H*_(5)_ = 17.7; *p* = 0.0034) and Dunn's multiple comparisons test in ***B***, Chi-square test (*χ*^2^_(2)_ = 1.98; *p* = 0.37) in ***C***, Mann–Whitney test in ***D*** (*U*_(2)_ = 271) and ***E*** (*U*_(2)_ = 173) and in ***H*** (*U*_(2)_ = 91), and Two-tailed Unpaired *t* test in ***G*** (*t*_(19)_ = 0.38) and in ***I*** (*t*_(15)_ = 0.43). Brackets beneath each dataset indicate numbers of cells (*n*).

Considering that ion channels are major modulatory targets of caldendrin (Tippens and Lee, 2007; Yang et al., 2018), we next tested whether MA currents were altered in DRGNs of *Cabp1* KO mice. In whole-cell patch clamp recordings, MA currents were elicited by cell pokes of varying displacement with a blunt glass probe. DRGNs exhibit complex combinations of MA currents which can be distinguished by their inactivation kinetics. As in previous studies (Hao and Delmas, 2010; Parpaite et al., 2021), we categorized MA currents in DRGNs according to their time constants of inactivation (*t*): rapidly adapting (*t* < 10 ms), intermediately adapting (10 ms < *t* < 30 ms), or slowly adapting (*t* > 30 ms; Fig. 3*A*). For each type of MA current, mean peak current amplitudes were significantly higher in *Cabp1* KO than in WT DRGNs (Fig. 3*A,B*). Current densities within each DRGN subclass were more variable in *Cabp1* KO than in WT mice, which could reflect the differences in caldendrin expression between DRGN subtypes based on the RNAseq and immunofluorescence analyses (Fig. 1; Lopez et al., 2023). There was no significant difference in the percentage of DRGNs in each category (Fig. 3*C*), which argued against the likelihood that the mechanical hypersensitivity of *Cabp1* KO mice resulted from a change in the representation of mechanosensory DRGN subtypes.

When averaged across DRGN subtypes, the indentation threshold was ∼20% lower and current density was ∼3-fold higher in *Cabp1* KO versus WT cultured DRGNs (Fig. 3*D,E*). In agreement with the expression of caldendrin in presumptive Ab-LTMRs (Fig. 1), the enhanced sensitivity and increased conductance of MA currents in *Cabp1* KO DRGNs were highest for medium- and large-DRGNs (i.e., capacitance >20 pF) and nominal for small DRGNs with capacitance characteristic of nociceptive DRGNs (<20 pF; Fig. 3*F*; Vilceanu and Stucky, 2010; Fan et al., 2011). Inactivation was slightly faster only for intermediately adapting (10 ms < *t* < 30 ms) currents (Fig. 3*G–I*), suggesting that loss of caldendrin differentially impacts the biophysical properties of MA channels in the distinct classes of DRGNs. Nevertheless, the lower threshold and higher current densities in *Cabp1 KO* compared to WT mice indicate a prominent role for caldendrin in suppressing MA currents in multiple DRGN subtypes including LTMRs.

**Figure 4.**
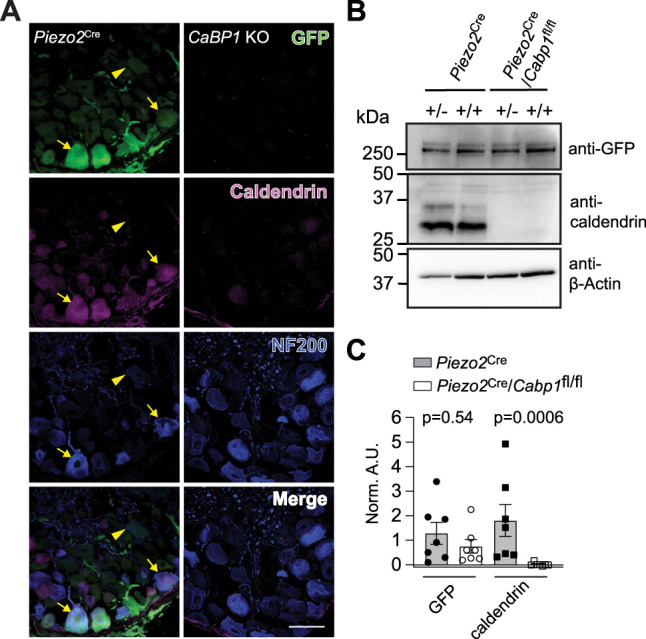
Characterization of *Piezo2*^Cre^/*Cabp1*^fl/fl^ mice. ***A***, Confocal micrographs showing DRG cryosections of *Piezo2*^Cre^ and *Cabp1* KO mice (left panels) labeled with antibodies against GFP, caldendrin, and NF200. Arrows indicate overlap label for GFP and caldendrin, arrowheads indicate DRGNs exhibiting signal for only GFP. Scale bar, 50 μm. Results are representative of 3 independent experiments (*N* = 3 mice). ***B,C***, Western blot analysis of DRG lysates from *Piezo2*^Cre^ and *Piezo2*^Cre^/*Cabp1*^fl/fl^ mice. Samples were obtained from mice that were heterozygous (+/−) or homozygous (+/+) for the *Piezo2*^Cre^ allele. Blots (***B***) were probed with antibodies against GFP, caldendrin, or β-Actin. Results are representative of 4 independent experiments (*N* = 4 mice). For densitometric quantification (***C***), signals corresponding to GFP-PIEZO2 and caldendrin from *Piezo2*^Cre^ (+/−) and *Piezo2*^Cre^ (+/+)/*Cabp1^fl/fl^* mice were normalized to that for β-Actin (Norm. A.U.); *p* values for the indicated comparisons were determined by Mann–Whitney test.

Multiple classes of MA channels are thought to give rise to the intermediately adapting and slowly adapting currents in DRGNs (Cho et al., 2002, 2006), but the genes corresponding to these channels remain unknown (Parpaite et al., 2021). However, the MA channel mediating the rapidly adapting currents has been unequivocally identified as PIEZO2 (Coste et al., 2010; Ranade et al., 2014) and numerous studies demonstrate a critical role for PIEZO2 in touch sensation and mechanical allodynia (Ranade et al., 2014; Murthy et al., 2018; Handler and Ginty, 2021; Handler et al., 2023). Thus, the tactile hypersensitivity of *Cabp1* KO mice could involve enhanced activity of PIEZO2. To address this possibility, we first determined whether depletion of caldendrin from PIEZO2-expressing DRGNs could phenocopy the tactile hypersensitivity of global *Cabp1* KO mice. For these studies, we bred a floxed *Cabp1* mouse strain (*Cabp1*^fl/fl^) with a *Piezo2*^Cre^ mouse strain in which a PIEZO2-GFP fusion protein and Cre recombinase are expressed under the endogenous PIEZO2 promoter (Woo et al., 2014). In *Piezo2*^Cre^ mice, caldendrin labeling coincided with GFP and NF200 labeling in numerous large-diameter DRGNs (Fig. 4*A*). Among NF200-positive DRGNs, ∼88% had labeling for both PIEZO2-GFP and caldendrin. Western blots of DRG lysates revealed bands corresponding to the caldendrin-S and caldendrin-L variants which are thought to arise from alternate start sites (see Fig. 6; Kim et al., 2014). These bands were present in control mice (*Piezo2*^Cre^) but not in mice with the conditional KO of *Cabp1* in *Piezo2*-expressing DRGNs (*Piezo2*^Cre^/*Cabp1*^fl/fl^, Fig. 4*B,C*).

**Figure 5.**
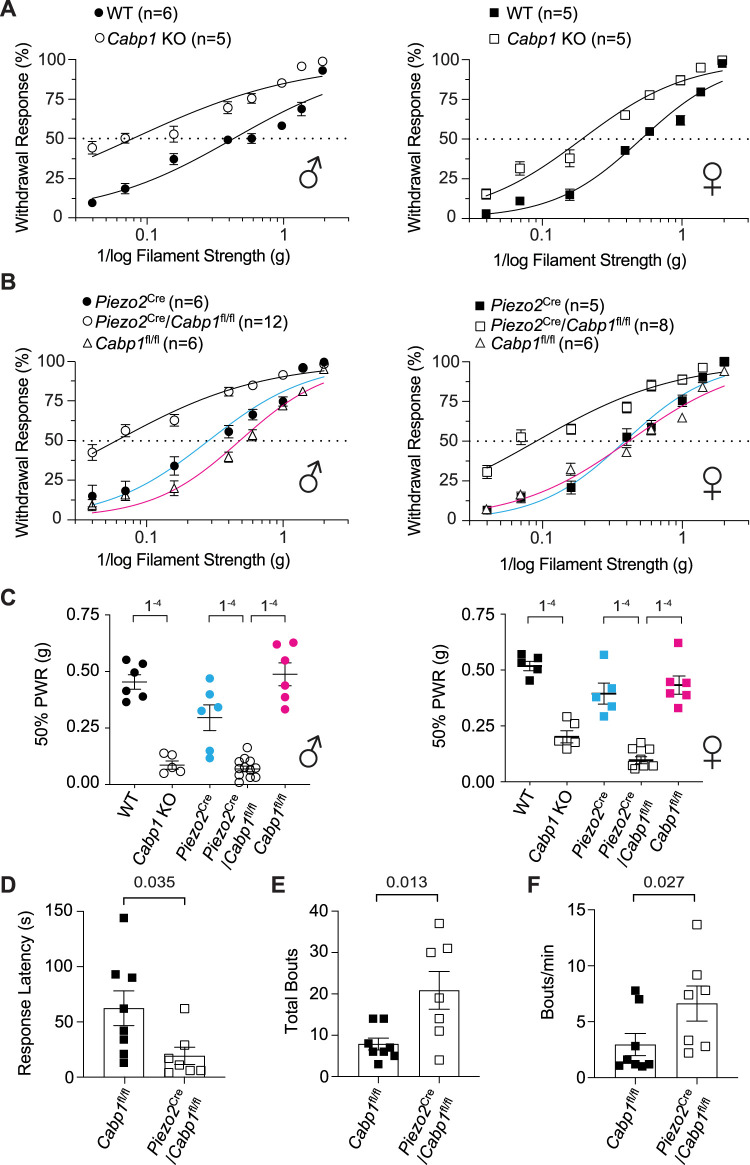
*Piezo2^Cre^*/*Cabp1^fl/fl^* exhibit enhanced mechanical sensitivity like *Cabp1* KO mice. ***A,B***, Von Frey assay was performed on WT versus Cabp1 KO (***A***) or Piezo2^Cre^/Cabp1^fl/fl^ versus *Piezo2^Cre^* or Cabp1^fl/fl^ mice (***B***) in males (left panels) and females (right panels). Withdrawal responses to 5 stimulus trials is plotted against 1/log filament strength. Symbols/bars represent mean ± SEM. Dotted line represents 50% withdrawal response. Smooth lines represent fit by non-linear regression. By 2-way ANOVA, datasets were significantly different for WT and *Cabp1* KO (*p* < 0.0001 for males, *p* < 0.0001 for females) and Piezo2^Cre^/Cabp1^fl/fl^ versus Piezo2^Cre^ (*p* < 0.0001 for males, *p* < 0.0001 for females) or Cabp1^fl/fl^ mice (*p* < 0.0001 for males, *p* < 0.0001 for females). *P* values for significantly different responses to specific filament strengths are summarized in Table 1. ***C***, Filament strength evoking half-maximal paw withdrawal response (50% PWR) is plotted from data in ***A*** and ***B*** for males (left panel) and females (right panel). Symbols represent responses from each animal. Bars represent mean ± SEM. Data were analyzed by one-way ANOVA: *F*_(4,30)_ = 36.37, *p* < 0.0001 for males, *F*_(4,24)_ = 34.88, *p* < 0.0001 for female data. Significant differences between groups (*p* < 0.0001, indicated above bars) were determined by Sidak's multiple comparisons test. There was no significant difference (relative to controls) for *Cabp1* KO (vs WT), *Piezo2*^Cre^/*Cabp1*^fl/fl^ (vs Cabp1^fl/fl^), and *Piezo*^Cre^/*Cabp1*^fl/fl^ (vs *Piezo2*^Cre^) for males (*F*_(2,21)_ = 1.08, *p* = 0.36) and females (*F*_(2,21)_ = 0.53, *p* = 0.59) by one-way ANOVA. ***D–F***, Tape response assays on separate cohorts of Piezo2^Cre^/Cabp1^fl/fl^ and control (Cabp1^fl/fl^) female mice measured in terms of behavioral responses (bouts) to tape application: the interval between tape application and first bout (response latency, ***D***), total number (***E***) and frequency (***F***) of bouts. *P* values for the indicated comparisons were determined by *t* test for (***D,E***) and Mann–Whitney test (***F***), *N* = 8 for Cabp1*^fl/fl^* and *N* = 7 for Piezo2^Cre^/Cabp1^fl/fl^ mice.

Next, we analyzed the mechanical sensitivity of the glabrous skin of *Piezo2*^Cre^/*Cabp1*^fl/fl^ mice in the von Frey assay. To enable rigorous comparisons with *Piezo2*^Cre^/*Cabp1*^fl/fl^ mice, we generated a parallel dataset involving additional cohorts of WT and global *Cabp1* KO mice. Like *Cabp1* KO mice, *Piezo2*^Cre^/*Ca*bp1^fl/fl^ mice were sensitive to the weakest filament strengths (Fig. 5*A,B*), and filament strengths evoking the half-maximal response were two to three times lower for *Piezo2*^Cre^/*Cabp1*^fl/fl^ mice than control mice (Fig. 5*C*). Except in female *Cabp1* KO mice, the increased responsiveness after global or conditional KO of caldendrin was highest for filament strengths ≤0.6 g (Table 1), suggesting an effect primarily on sensitivity to nonnoxious mechanical stimuli. Moreover, there was no significant difference in the heightened withdrawal responses of the global and conditional *Cabp1* KO mice (Table 1). To test whether loss of caldendrin impacted mechanical sensitivity in hairy skin, we used an assay in which mice are tested for the ability to remove a piece of tape from their back (Ranade et al., 2014; Murthy et al., 2018; Michel et al., 2020). In these experiments, *Piezo2*^Cre^/*Cabp*1^fl/fl^ was quicker to respond to the applied tape than control mice (CaBP1^fl/fl^, Fig. 5*D*). *Piezo2*^Cre^/*Cabp*1^fl/fl^ also exerted more effort in removing the tape in that their total responses and responses/min were higher than control mice (Fig. 5*E,F*). These results indicate that tactile hypersensitivity in glabrous and hairy skin in *Cabp1* KO mice stems from defects in PIEZO2-expressing DRGNs.

**Table 1.** Statistical comparisons of withdrawal responses (%) in von Frey assay

	Males		Females	
Filament strength (g)	Mean difference (%)	*p* value	Mean difference (%)	*p* value
WT vs *Cabp1* KO				
0.04	−35.67	<0.0001	−12.4	0.0231
0.07	−32.7	<0.0001	−20.6	<0.0001
0.16	−16.13	0.0039	−23	<0.0001
0.40	−20.83	<0.0001	−22.4	<0.0001
0.60	−26	<0.0001	−23	<0.0001
1.00	−27.67	<0.0001	−25.4	<0.0001
1.40	−27.63	<0.0001	−15.4	0.0022
2.00	−5.833	0.8161	−2	0.9996
*Piezo2*^Cre^ vs *Piezo2*^Cre^/*Cabp1*^fl/fl^				
0.04	−27.67	<0.0001	−24.03	<0.0001
0.07	−38.17	<0.0001	−37.9	<0.0001
0.16	−28.92	<0.0001	−36.7	<0.0001
0.40	−25.42	<0.0001	−18.65	0.002
0.60	−18.58	0.004	−26.2	<0.0001
1.00	−16.83	0.0124	−13.35	0.0588
1.40	0.4167	>0.9999	−6.05	0.8608
2.00	1.25	>0.9999	0	>0.9999
*Cabp1*^fl/fl^ vs *Piezo2*^Cre^/*Cabp1*^fl/fl^				
0.04	−34.33	<0.0001	−23.96	<0.0001
0.07	−41.5	<0.0001	−36.67	<0.0001
0.16	−43.92	<0.0001	−25.83	<0.0001
0.40	−42.08	<0.0001	−28.75	<0.0001
0.60	−31.92	<0.0001	−28.33	<0.0001
1.00	−19.33	0.0007	−24.58	<0.0001
1.40	−14.58	0.021	−12.92	0.0319
2.00	−2.917	0.998	−6.667	0.677

Mean differences in % withdrawal responses for control and *Cabp1* KO strains are listed for the individual filament strengths for data shown in Figure 5*A,B*. *P* values were determined by Tukey's multiple comparison test with a single pool variance. There was no significant difference in mean difference in % withdrawal responses (relative to controls) for *Cabp1* KO (vs WT), *Piezo2*^Cre^/*Cabp1*^fl/fl^ (vs Cabp1^fl/fl^), and *Piezo*^Cre^/*Cabp1*^fl/fl^ (vs *Piezo2*^Cre^) for males (*p* = 0.36, *F*_(2,21)_ = 1.08) and females (*p* = 0.59, *F*_(2,21)_ = 0.53) by one-way ANOVA.

### Caldendrin interacts with and inhibits PIEZO2

**Figure 6.**
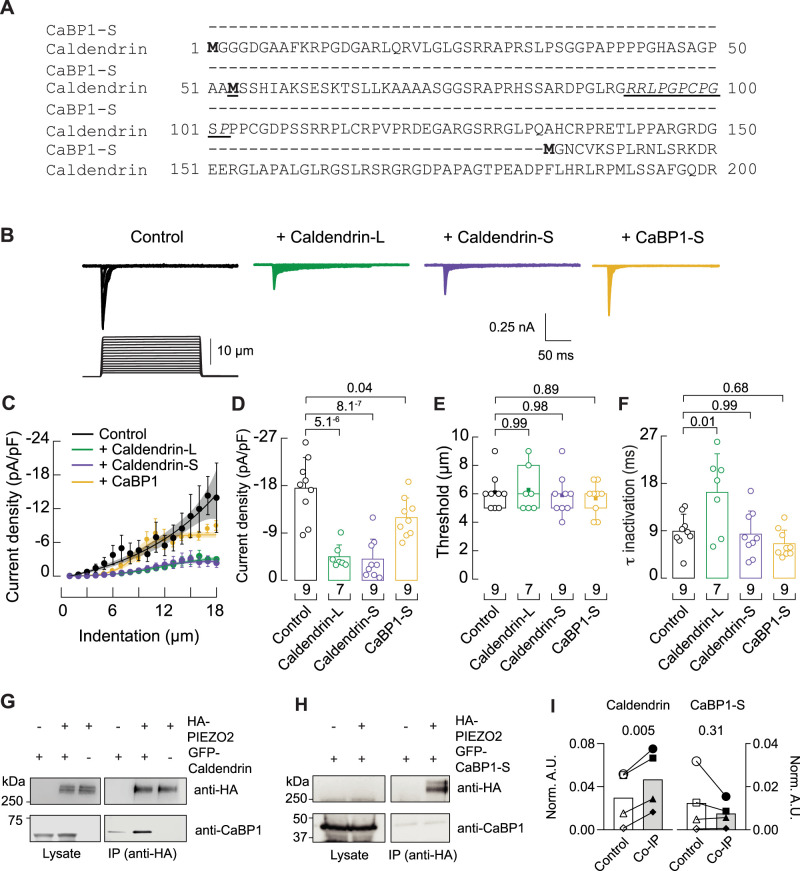
Caldendrin inhibits and interacts with PIEZO2. ***A***, Amino acid (AA) sequence alignment of mouse sequences corresponding to divergent N-terminal domain of CaBP1-S (Genbank #AAF25787.1) and Caldendrin (Genbank #AHX83451.1). In bold are alternative start sites for caldendrin-L and caldendrin-S (bold + underline), as well as starting methionine of CaBP1-S. PxxP motifs are indicated in underlined italics. ***B***, representative currents traces evoked by mechanical indentation at −60 mV in whole-cell patch-clamp recordings of Piezo1-deficient N2A cells transfected with PIEZO2 alone (control) or co-transfected with caldendrin-L, caldendrin-S, or CaBP1. ***C***, Current density responses to various displacements; a Boltzmann function was fitted to the data. ***D–F***, mean current densities (***D***), displacement thresholds (***E***), one-way ANOVA, df1 = 3, df2 = 30; *F* = 0.32; *p* = 0.81 and Tukey test), and time constant (*t*) for inactivation. *P* values were determined by Kruskal-Wallis and Dunn's Multiple Comparison Test (*H*_(3)_ = 22.5; *p* = 5.04^−5^ in ***D***; *H*_(3)_ = 8.9; *p* = 0.031 in ***F***), and one-way ANOVA and Tukey’s test in ***E*** (*F*_(3,2)_ = 0.32; *p* = 0.81). Boxplots show mean (square), median (bisecting line), bounds of box (75th to 25th percentiles), and minimum and maximum data points. Bars are mean ± SD. Number of cells is denoted below columns. ***G,H***, Western blots showing co-immunoprecipitation (Co-IP) of PIEZO2 with caldendrin (***G***) but not CaBP1-S (***H***). HEK293T cells were transfected with HA-tagged PIEZO2, GFP-tagged caldendrin or CaBP1, alone or together. Western blots were probed with antibodies against HA, CaBP1/caldendrin, or b-actin. Left panels, lysate (20% of input for IP); right panels, IP samples. Results are representative of four independent experiments. ***I***, Densitometric quantification of signals (Norm. A.U.) corresponding to GFP-caldendrin or GFP-CaBP1-S co-IP'ed with anti-HA antibodies from cells transfected with GFP-Caldendrin or GFP-CaBP1-S alone (Control) or co-transfected with HA-PIEZO2 (Co-IP). Results represent 4 independent transfections; *p* values were determined by paired *t* test.

Tactile hypersensitivity could result from the enhanced activity of MA channels such as PIEZO2, in *Cabp1* KO DRGNs. Given that multiple channels give rise to MA currents in DRGNs (Ranade et al., 2014; Chesler et al., 2016; Parpaite et al., 2021; Handler et al., 2023), we isolated the effects of caldendrin on PIEZO2 in electrophysiological analyses of neuro2A (N2A) cells transfected with their corresponding cDNAs. To eliminate the contribution of PIEZO1, which is endogenously expressed in N2A cells, we used a clonal N2A cell line in which PIEZO1 was knocked out (Moroni et al., 2018). In agreement with the increase in MA current density upon loss of caldendrin from DRGNs (Fig. 3), PIEZO2-mediated current densities were significantly lower upon co-transfection with either caldendrin-S or caldendrin-L variant (Fig. 6*B–D*). However, displacement thresholds were not affected (Fig. 6*E*) and co-transfection of PIEZO2 with caldendrin-L but not caldendrin-S caused a slowing of inactivation (Fig. 6*F*).

Caldendrin and CaBP1 variants contain EF-hand Ca^2+^ binding domains, the second of which contains amino acid substitutions that disrupt Ca^2+^ binding (Haeseleer et al., 2000; Wingard et al., 2005; Fig. 6*A*). Unlike CaBP1, caldendrin has a bipartite structure in which a unique N-terminal domain contains proline rich (PxxP) motifs known to bind to SH3 domains (Fig. 6*A*; Mundhenk et al., 2019). To gain insight into the molecular determinants underlying the regulation of PIEZO2 by caldendrin, we compared the effects of co-transfecting PIEZO2 with caldendrin or CaBP1 in PIEZO1-deficient N2A cells. In contrast to caldendrin-L and caldendrin-S, CaBP1 did not significantly suppress PIEZO2 current density or slow inactivation kinetics (Fig. 6*B–F*). To test whether the regulation of PIEZO2 could involve a physical association with caldendrin, we analyzed whether the two proteins could be co-immunoprecipitated. When co-transfected with GFP-tagged caldendrin, hemagglutinin (HA) antibodies pulled down HA-tagged PIEZO2 and GFP-caldendrin (Fig. 6*G*). In contrast, GFP-tagged CaBP1 did not co-immunoprecipitate with HA-PIEZO2 (Fig. 6*H,I*). These results show that caldendrin interacts with and modulates PIEZO2 in a manner that is not reproduced by CaBP1.

**Figure 7.**
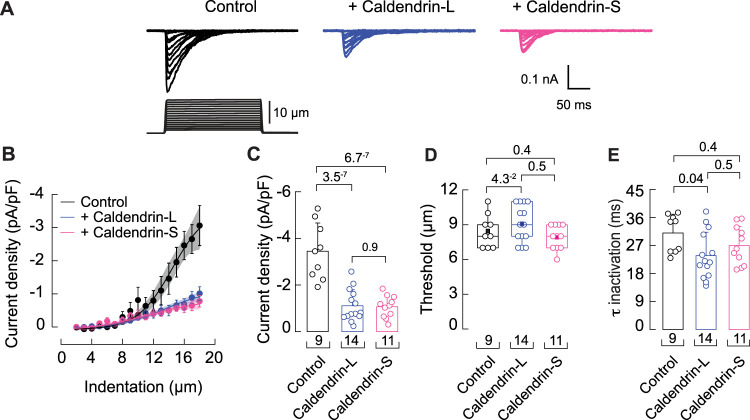
Caldendrin inhibits PIEZO1 in N2A cells. ***A***, Representative traces for currents elicited by mechanical stimulation in N2A cells transfected with caldendrin-L or -S. ***B–E***, Current density responses to various displacements (***B***), symbols represent mean ± SEM; highlighted area represents the 95% confidence band for each curve; a Boltzmann function was fitted to the data); mean current densities (***C***), bars are mean ± SD; one-way ANOVA, *F*_(2,31)_ = 28.35; *p* = 9.98^−8^ and Tukey’s test), displacement thresholds (***D***), boxplots show mean (square), median (bisecting line), bounds of box (75th to 25th percentiles), outlier range with 1.5 coefficient (whiskers), and minimum and maximum data points. One-way ANOVA (*F*_(2,31)_ = 2.17; *p* = 0.13) and Tukey’s test), and time constant (*t*) for inactivation (***E***), bars are mean ± SD; one-way ANOVA (*F*_(2,31)_ = 3.21; *p* = 0.05) and Tukey’s test.). *n* is denoted in each panel. Post hoc *p* values are denoted above the bars.

To determine if the regulation by caldendrin extended to the PIEZO1 subtype, we analyzed the effects of transfecting caldendrin variants in N2A cells in which the endogenous expression of PIEZO1 was not eliminated (Coste et al., 2010). PIEZO1 mediated currents were significantly inhibited upon transfection with caldendrin-S or caldendrin-L (Fig. 7*A–C*). While displacement thresholds were not affected (Fig. 7*D*), co-transfection with caldendrin-L caused a modest yet significant acceleration of inactivation kinetics (Fig. 7*E*). Thus, while acting as an overall suppressor of MA channels, caldendrin exerts slightly distinct modulatory effects on PIEZO1 and PIEZO2.

## Discussion

In this study, we show that caldendrin is needed to control touch sensation and cellular responses to mechanical stimuli. In the absence of caldendrin, mice exhibit tactile hypersensitivity and MA current densities are increased in DRGNs. Our analyses of mice with conditional KO of caldendrin in *Piezo2*-expressing DRGNs and of PIEZO2 in transfected N2A cells implicate PIEZO2 as one of the MA channels subject to inhibitory modulation by caldendrin. While the role of Ca^2+^ sensor proteins as integral modulators of other ion channels is well-established, our study provides the first evidence for this type of regulation of MA channels in peripheral sensory neurons. Our findings add caldendrin to a growing list of protein partners that could serve as modulators of PIEZO2 and potentially other MA channels (Narayanan et al., 2016).

### Caldendrin as a negative regulator of MA channels

Electrophysiological analyses of DRGNs indicate the presence of multiple subtypes of MA channels that differ in terms of their biophysical properties and modes of activation (Hu and Lewin, 2006; Hao and Delmas, 2010; Parpaite et al., 2021; Murthy, 2023). While PIEZO2 accounts for most of the rapidly adapting and some intermediately adapting MA currents (Ranade et al., 2014; Murthy et al., 2018), the channels that mediate the other classes of MA currents remain unknown. Our findings that the average current densities were increased in *Cabp1 KO* DRGNs regardless of their inactivation kinetics (Fig. 3*A,B*) implies a modulatory role for caldendrin that is shared among different MA channel subtypes. Given that caldendrin binds to components or modifiers of the cytoskeleton (e.g., cortactin (Mikhaylova et al., 2018), myosin V (Konietzny et al., 2022), MAP1A (Seidenbecher et al., 2004)), caldendrin could act as a brake on cytoskeletal forces needed for mechanogating (Cox et al., 2019; Verkest et al., 2022). Loss of this brake could produce the lower indentation threshold for MA currents in *Cabp1* KO DRGNs (Fig. 3*D*). Concurrently, an interaction of caldendrin with myosin V could place a limit on the cell surface density of MA channels. Caldendrin inhibits the processive movement of myosin V (Konietzny et al., 2022), and this impairs the transport of AMPA receptors through endosomes to the cell surface (Sumi and Harada, 2020). In a similar fashion, caldendrin could restrict the number of functional MA channels by retarding their forward trafficking.

### Caldendrin as a negative regulator of PIEZO2

In addition to the types of stimuli to which they respond, mechanosensory DRGNs are classified according to their force thresholds, conduction velocities, and rates of adaptation. Whereas higher-intensity forces activate HTMRs involved in the transmission of painful mechanical stimuli, gentle forces enable the discrimination of textures and shapes through the recruitment of LTMRs (Handler and Ginty, 2021). The increased MA currents with decreased mechanical thresholds in *Cabp1* KO DRGNs provide a logical mechanism for the enhanced tactile sensitivity of *Cabp1* KO and *Piezo2^Cre^*/*Cabp1^fl/fl^* mice (Figs. 3, 5). The interpretation that PIEZO2-mediated currents are enhanced in *Cabp1* KO DRGNs is supported by our findings that caldendrin-S and caldendrin-L profoundly reduced PIEZO2-mediated currents in N2A cells (Fig. 3*B–D*). However, caldendrin did not affect the indentation threshold of PIEZO2 in N2A cells, as predicted by results in *Cabp1* KO DRGNs (Figs. 3*D*, 6*E*). Moreover, inactivation of PIEZO2 was slowed by caldendrin-L in N2A cells but was unchanged for rapidly inactivating MA currents in *Cabp1* KO DRGNs (Figs. 3*G*, 6*F*). The latter discrepancy could be explained by the fact that caldendrin-S does not affect PIEZO2 inactivation (Fig. 6*F*) and so could neutralize the impact of caldendrin-L on inactivation in DRGNs where both variants are expressed (Fig. 4*B*; Lopez et al., 2023). In addition, N2A cells exhibit properties of transformed cells and may not fully recapitulate the cellular context of DRGNs.

Our coimmunoprecipitation results (Fig. 6*G–I*) raise the intriguing possibility that the effects of caldendrin on PIEZO2 could be mediated by a direct protein interaction as is the case for other ion channels. CaBP1 and caldendrin oppose CaM-dependent inactivation of Ca_v_1 L-type Ca^2+^ channels by competing with CaM for binding to the channel (Zhou et al., 2004, 2005). In a manner that is not replicated by CaM, CaBP1 binds to and inhibits inositol trisphosphate receptors (IP_3_Rs) (Haynes et al., 2004; Kasri et al., 2004; Li et al., 2009) and TRPC5 channels (Kinoshita-Kawada et al., 2005). However, we cannot rule out that the association of caldendrin with PIEZO2 is due to tertiary protein interactions. The N-terminal domain of caldendrin binds to cortactin (Mikhaylova et al., 2018) and myosin V (Konietzny et al., 2022) and these proteins could subsequently interact with PIEZO2 and other MA channels. Notably, the N-terminal domain of caldendrin-L is absent in CaBP1 and is modified in the caldendrin-S variant. These differences could explain the differences in the effects of caldendrin-S and caldendrin-L on inactivation and the inability of CaBP1 to coimmunoprecipitate with or significantly modulate PIEZO2 channels (Fig. 6). Additional studies are required to map the molecular determinants and possibly additional protein interactions that link caldendrin in the MA channel complex.

The similar effects of caldendrin on PIEZO1 and PIEZO2 current density in N2A cells suggest that the molecular determinants which enable inhibitory regulation by caldendrin are conserved in both PIEZO subtypes. However, the effect of caldendrin-L in slowing inactivation of PIEZO2 was not reproduced in PIEZO1 (Figs. 6*F*, 7*E*). Perhaps the inherently slower inactivation of PIEZO1 compared to PIEZO2 (Coste et al., 2010) may occlude any modulatory effect of caldendrin in this regard. Comparative analysis of how caldendrin differentially modifies the kinetics of PIEZO1 and PIEZO2 could yield insights into the biophysical mechanism that distinguishes the PIEZO subtypes in this regard. Recent findings indicate that PIEZO1 mediates intermediately adapting MA currents in DRGNs and regulates itch sensation (Hill et al., 2022). Thus, it is intriguing to speculate that the MA currents that were upregulated in intermediately adapting DRGNs in *Cabp1* KO mice (Fig. 7*A,B*) could be mediated in part by PIEZO1.

While we did not investigate itch behaviors, our studies show that *Cabp1* KO mice lack a proprioceptive defect even though caldendrin is expressed in proprioceptors (Figs. 1*C*, 2*D*). This was somewhat surprising considering the severe proprioceptive impairment in conditional *Piezo2* KO mice (Woo et al., 2015). However, it is not clear whether a gain of function in MA currents in DRGNs from mature mice would be expected to cause proprioceptive defects. In knock-in mice expressing a gain-of-function *Piezo2* mutation, joint defects and proprioceptive impairments are caused by aberrant mechanosensation during early development (embryonic day 12.5 or at P7–10) but not after P21 or during adulthood (Ma et al., 2023). In the mouse brain and cochlea, CaBP1 expression levels are relatively low during the first postnatal week and increase to adult levels in the second 2 weeks (Laube et al., 2002; Yang et al., 2016). If the same is true in DRGNs, *Cabp1* KO mice may lack a phenotype similar to *Piezo2* knock-in mice because caldendrin is not a significant modulatory factor during the developmental window in which excessive PIEZO2 activity derails limb formation (Ma et al., 2023). Alternatively, caldendrin may not be targeted to distal regions of afferent nerve endings in the muscle and Golgi tendon organs where PIEZO2 actions on proprioception may be most prominent.

### Physiological relevance of caldendrin in mechanical sensitivity

The augmentation of PIEZO2-mediated currents in *Cabp1* KO DRGNs is expected to boost the stimulus response properties of Ab-LTMRs, possibly by increasing their firing rate and lowering their mechanical thresholds (Ranade et al., 2014). However, caldendrin could also regulate PIEZO2 function in non-neuronal cell types such as Merkel cells which form specialized complexes with Ab-LTMRs in the skin (Woo et al., 2014). We also do not discount the possibility that caldendrin could affect the firing properties of Ab-LTMRs through effects on targets in addition to MA channels. Like CaM, caldendrin is expected to interact with multiple protein partners and could regulate the expression and or function of a variety of ion channels (Tippens and Lee, 2007; Dieterich et al., 2008). Elucidating the mechanisms whereby caldendrin may impact DRGN excitability is complicated by the functional diversity of Ab-LTMRs, with each subtype expressing a distinct array of ion channels that control DRGN firing properties (Zheng et al., 2019). The use of *Cabp1*^fl/fl^ mice with appropriate Cre driver lines would help pinpoint the role of caldendrin in different cell types of the touch sensation circuitry.

An important follow-up question centers around the need for inhibitory modulation of tactile sensitivity. A physiological brake on PIEZO2 function may help fine-tune mechanical sensitivity in various contexts. For example, cutaneous sensitivity to mechanical stimuli in mice declines with age in parallel with a decrease in PIEZO2-mediated currents in DRGNs, despite no changes in levels of *Piezo2* mRNA (Michel et al., 2020). Since caldendrin undergoes a developmental upregulation in the brain and cochlea (Laube et al., 2002; Yang et al., 2016), it is tempting to speculate that a similar increase in caldendrin in DRGNs could lead to the maturational decline in touch sensation. A second possibility relates to the gate control theory of pain, which posits that LTMR-mediated touch signals inhibit transmission of mechanical pain by HTMRs via activation of spinal inhibitory interneurons (Melzack and Wall, 1965). This mechanism can explain why gentle touch alleviates acute pain in humans and is supported by findings that optogenetic stimulation of Ab-LTMRs suppresses mechanically evoked pain in mice (Arcourt et al., 2017). Conversely, increasing MA currents through ectopic expression of PIEZO1 in all DRGNs suppresses mechanical pain in mice (Zhang et al., 2019). The basal inhibition of PIEZO2 by caldendrin may dampen LTMR input to maintain the sensitivity of HTMRs to acutely harmful stimuli. Future studies aimed at exploring mechanical pain sensitivity in *Cabp1* KO mice could reveal the disruption of caldendrin/PIEZO2 interactions in LTMRs as a novel strategy to lessen pain.
